# Platinum-Based Drugs Differentially Affect the Ultrastructure of Breast Cancer Cell Types

**DOI:** 10.1155/2017/3178794

**Published:** 2017-03-09

**Authors:** Shadia Al-Bahlani, Buthaina Al-Dhahli, Kawther Al-Adawi, Abdurahman Al-Nabhani, Mohamed Al-Kindi

**Affiliations:** ^1^Department of Allied Health Sciences, College of Medicine and Health Sciences, Sultan Qaboos University, Muscat, Oman; ^2^Department of Pathology, College of Medicine and Health Sciences, Sultan Qaboos University, Muscat, Oman

## Abstract

Breast cancer (BC) is the most common cause of cancer-related death worldwide. Although platinum-based drugs (PBDs) are effective anticancer agents, responsive patients eventually become resistant. While resistance of some cancers to PBDs has been explored, the cellular responses of BC cells are not studied yet. Therefore, we aim to assess the differential effects of PBDs on BC ultrastructure. Three representative cells were treated with different concentrations and timing of Cisplatin, Carboplatin, and Oxaliplatin. Changes on cell surface and ultrastructure were detected by scanning (SEM) and transmission electron microscope (TEM). In SEM, control cells were semiflattened containing microvilli with extending lamellipodia while treated ones were round with irregular surface and several pores, indicating drug entry. Prolonged treatment resembled distinct apoptotic features such as shrinkage, membrane blebs, and narrowing of lamellipodia with blunt microvilli. TEM detected PBDs' deposits that scattered among cellular organelles inducing structural distortion, lumen swelling, chromatin condensation, and nuclear fragmentation. Deposits were attracted to fat droplets, explained by drug hydrophobic properties, while later they were located close to cell membrane, suggesting drug efflux. Phagosomes with destructed organelles and deposits were detected as defending mechanism. Understanding BC cells response to PBDs might provide new insight for an effective treatment.

## 1. Introduction

Breast cancer is the most common cause of cancer-related deaths in women worldwide [1]. It is characterized by heterogeneity as it exhibits wide scope of morphological features, different immunohistochemical profiles, and unique histopathological subtypes. Breast cancer can be classified according to immunohistochemical phenotypes [i.e., presence or absence of estrogen receptor (ER), progesterone receptor (PgR), and epidermal growth factor receptor 2 (HER2)] into five subtypes. These are luminal A, luminal B, HER2 overexpression, basal-like, and normal-like subtypes, each of which has distinct clinical outcome [2]. Luminal A accounts for 50% of invasive breast cancers. It is ER/PgR positive or HER2 negative. Luminal B category represents 20% of invasive breast cancers. The ER/PgR is positive, while HER2 expression is variable (positive or negative). HER2 overexpression group accounts for 15% of all invasive breast cancers. The ER/PR is usually negative. The basal class is typically ER/PR negative, and HER2 negative (triple-negative). It comprises about 15% of all invasive breast cancers. It has generally poor prognosis. Normal-like tumors account for 7.8% of all breast cancer cases in a lymph-node negative cohort. It is positive for ER and PgR but negative for HER2 [3, 4].

Due to this heterogeneity in breast cancer, the treatment is complicated and the therapeutic strategies should be chosen carefully. To overcome the disease, each patient should be treated individually according to the morphological classification with molecular parameters and sensitivity to available therapy. Systemic therapy, including chemotherapy, endocrine therapy, and targeted treatments, have markedly reduced the risk for recurrence and mortality after primary treatment of breast cancer and have increased the 5- and 10-year survival rates [5].

Adjuvant chemotherapy termed platinum-based drugs (PBDs), such as Cisplatin, Carboplatin, and Oxaliplatin, are important effective drugs used for various cancer types. Platinum-DNA adducts, which are formed following uptake of the drug into the nucleus of cells, activate several cellular processes such as DNA-damage recognition and repair, cell-cycle arrest, and programmed cell death/apoptosis processes that mediate the cytotoxicity of these platinum drugs [6, 7]. Cisplatin (cis-diamminedichloroplatinum (II)) is the first generation of PBDs used as anticancer. Cisplatin induces dose-limiting toxicity causing some side effects including nephrotoxicity, ototoxicity, and nausea and vomiting. In order to overcome this, Carboplatin (cis-diammine-1,1-cyclobutane dicarboxylate platinum (II)) was developed and it is considered as a second generation of PBDs. However this drug has lower reactivity compared to Cisplatin but it is suitable for aggressive high-dose chemotherapy. Cisplatin and Carboplatin developed resistance in some of the cancers, the reason why Oxaliplatin was developed. It is a platinum complex with (1R,2R)-1,2-diaminocyclohexane (DACH) ligand and oxalate as a leaving group. The toxic side effect of this drug is significantly reduced due to oxalate group which lowers its reactivity [8].

The PBDs have been used for 3 decades in many types of cancers including ovarian, cervical, head and neck, and non-small-cell lung cancer [9–11]. However, the use of PBDs for breast cancer in clinical practice is not common, except for BRACA-1 deficient breast cancer and triple-negative breast cancer [12]. Although they are initially effective, their efficacy is limited by the occurrence of resistance which is attributed to alterations in cellular pathways such as DNA repair, drug transport and metabolism, and apoptosis. In order to understand the mechanism of PBDs resistance, many studies explore the role of these pathways and their interaction at both cellular and molecular levels [13, 14]. Having said that, not many studies assess such role in breast cancer since these drugs are not routinely used. For that reason, the current study aimed to assess the effect of PBDs and their ultrastructural alterations on the intracellular organelles of breast cancer cells. Three models of breast cancers, each of which has distinct immunohistochemical profile, were used to examine such effects. The MCF-7 cell line representing the luminal A breast cancer is positive for ER and PgR but negative for HER2 [15] while BT-474 cell line is a luminal B tumor and positive for all the three receptors [16]. Luminal B tumors have higher proliferation and poorer prognosis than luminal A tumors. Finally, the MDA-MB-231 cell line was used to represent the basal-like subtype as the triple-negative breast cancer that lacks all the three receptors [17]. The first two cell lines are more differentiated and therefore more susceptible to the used treatments unlike the later which is difficult to treat with poor prognosis [18].

Each cell line was treated with different concentrations of Cisplatin, Carboplatin, and Oxaliplatin and their effects were detected at different timing. The effects of these drugs on the surface and the ultrastructural features of each cell types were detected by SEM and TEM.

To our knowledge we are the first to show a comprehensive comparison of cellular features of representative breast cancer cells using electron microscope. We are also the first to detect the distinctive effects of three commonly used PBDs on these cells and how these effects might differ from one cell to another depending on the cell type and drug action. Our findings will help and direct further investigations to pursue specific pathways that might be involved in determining the breast cancer cell sensitivity to PBDs.

## 2. Materials and Methods

### 2.1. Reagents

Fetal bovine serum (FBS), hexamethyldisilazane (HMDS), and phosphate buffer saline were obtained from SIGMA-ALDRICH, USA. Roswell Park Memorial Institute medium (RPMI), Dulbecco's Modified Eagle Medium (DMEM), 1% penicillin and streptomycin, and 0.25% Trypsin-EDTA were purchased from Gibco, USA. Sodium cacodylate buffer, glutaraldehyde fixative, 1% osmium tetroxide, Resin Toluidine Blue, uranyl acetate, lead citrate, and Al stubs were obtained from Agar Scientific, UK, and acetone (25%, 50%, 75%, 95%, and 100%) was obtained from Fisher Scientific, UK.

### 2.2. Cell Culture

Human breast cancer cell lines, MDA-MB-231, MCF-7, and BT-474, were purchased from National Cell Bank of Iran (NCBI) and recently used by Hooshmand et al. and Muhammadnejad et al. [19, 20] while normal epithelial breast cells (MCF 10A) were a generous gift from Professor Allal Ouhtit, University of Qatar [21]. Both MDA-MB-231 and MCF-7 were cultured in Dulbecco's Modified Eagle Medium (DMEM) while BT-474 was in Roswell Park Memorial Institute media (RPMI 1640). MCF-10A cell line was propagated in Ham's DMEM-F12 (1 : 1 dilution) with 2.5 mM L-glutamine, 20 *μ*g/mL epidermal growth factor, 0.1 *μ*g/mL cholera toxin, and 5% horse serum. All media were supplemented with 10% fetal bovine serum, 1% penicillin, and 1% streptomycin. All cell lines were adherent and propagated in a humidified incubator at 37°C with 5% CO_2_ atmosphere. For treatment, cells were seeded into 6-well plates till they reach 80% confluency. They were treated with different concentrations (0, 10, and 20 *μ*m) of Cisplatin, Carboplatin, and Oxaliplatin at different time points (0.5, 2, 4, and 12 hours). Posttreated cells were detached by 0.25% trypsin-EDTA and complete media were added to neutralize the trypsin. Cells were centrifuged at 200*g* for 5 minutes. The supernatant was discarded and the pellet was resuspended in PBS and centrifuged at 200*g* for 3.5 minutes to be ready for further processing.

### 2.3. Scanning Electron Microscopy (SEM)

Cells were prepared for SEM as described earlier [22]. Briefly, they were cultured on cover-slips and treated as mentioned above. They were then harvested and fixed in 2.5% glutaraldehyde for 12 hours and then washed twice with cacodylate buffer, each wash for 5 minutes. They were postfixed with 1% osmium tetroxide for 1 hour, dehydrated using gradual concentrations of ethanol (25%, 75%, 95%, and 99.9%), and then dried using hexamethyldisilazane (HMDS). Subsequently, they were mounted on Al stubs and coated with gold particles. Micrographs were revealed using JEOL JSM-5600LV scanning electron microscope.

### 2.4. Transmission Electron Microscopy (TEM)

Cells were prepared for TEM as described earlier [22]. Briefly, PBS-washed cells were fixed in glutaraldehyde and kept on ice for 1 hour. Cells were then rinsed twice for 5 minutes with isotonic buffer (0.1 M sodium cacodylate buffer, pH 7.2–7.4). The second fixative, 1% osmium tetroxide, was added to the cells for 1 hour followed by rinsing cells twice with distilled water, each for ten minutes. Fixed cells were dehydrated in serial dilutions of acetone (25%, 50%, 75%, and 95%), 10 minutes in each concentration. Cells were then dehydrated completely in 3 changes of absolute acetone, 10 minutes each. After that, infiltration was performed by placing the cells in a mixture of acetone and resin in a ratio of 1 : 1 for 1 hour and then with a ratio of 1 : 3 for 30 minutes to gradually replace the acetone with the supporting medium (resin). Pure resin was then added for 1 hour to remove traces of acetone. Cells were embedded in pure resin which was polymerized at 60°C oven overnight. Sections were produced at thickness of 0.5 *μ*m to check availability of cells in the block by light microscope. Sections were then stained with Toluidine Blue for 1 minute and examined under the light microscope. Ultrathin sections (70 nm thick) were then produced and placed on a copper grid. The grids were floated over a drop of uranyl acetate for 30 minutes. Then, cells were washed in 50% ethanol followed by distilled water. Grids were then floated over a drop of lead citrate for 30 minutes. Cells were washed in distilled water and allowed to dry over a filter paper. Micrographs were screened by transmission electron microscope JEOL JEM-1230.

## 3. Results

### 3.1. Breast Cancer Cells Exhibited Distinct Features among Them

**Figure 1:**
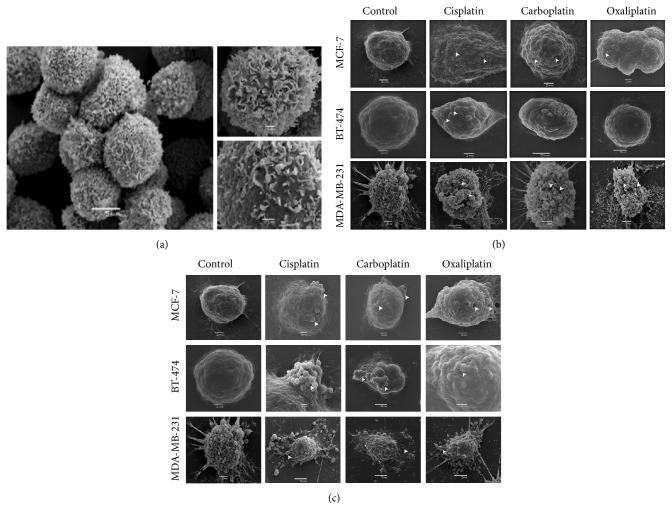
Representative SEM micrographs of breast cell lines. (a) Untreated normal breast cell line (MCF-10A). Cells are in contact with each other featured by short lamellipodia. (b) Breast cell lines (MCF-7, BT-474, and MDA-MB-231) treated with three types of PBDs, Cisplatin, Carboplatin, and Oxaliplatin, at 15-minute interval in comparison to the controlled group. Surface pores are illustrated by head arrow. Micrographs are screened at scale of 2–5 *μ*M. (c) Breast cell lines (MCF-7, BT-474, and MDA-MB-231) treated with three types of PBDs, Cisplatin, Carboplatin, and Oxaliplatin, in comparison to the controlled group at 12-hour interval. Grooves and apoptotic features are indicated by head arrow. Micrographs are screened at scale of 1–10 *μ*M.

SEM provides high magnifications of tested cells that might provide possible morphofunctional correlations. In order to first describe the 3D structure of each cell, SEM was used to scan breast cancer cells, MCF-7, BT-474, and MDA-MB-231, without any treatment and point out the similarities and differences among them. SEM micrograph (Figure 1(b)) of the nontreated cells revealed a semiflattened surface structure containing microvilli with extending lamellipodia known as membrane ruffles. These ruffles were less in number and finer in shape for both MCF-7 and BT-474 cells while MDA-MB-231 cells showed higher numbers and thicker membrane-bound protrusions and lamellipodia. In contrast, the normal breast cells, MCF-10A, are round in shape and characterized by short lamellipodia as it is shown in Figure 1(a).

### 3.2. PBDs Caused Pores and Early Apoptotic Shape Modifications

SEM also provides the qualitative observations of PBDs-dependent alterations and therefore our next step was to detect these morphological alterations at two time points (15 minutes and 12 hours) using two concentrations of 10 and 20 *μ*M. PBDs have quick action on the cancer cells, the reason why we choose 15-minute time point. At early stage of treatment, all cell types responded by forming cell membrane specializations represented in various patterns (Figure 1(b)), one of which is the formation of pores allowing the drug to enter the cell. Another important pattern was featured by shrunken and round-shaped cells, indicated by the lamellipodia retraction. The prolonged treatment after 12 hours revealed the early stage of apoptosis presented by convoluted membrane, membrane blebs, and apoptotic bodies. The membrane blebbing is considered to be a specific pattern of apoptosis that is due to a deep cytoskeleton rearrangement, resulting in alterations of cell shape and organelles distribution as will be illustrated later in TEM micrographs. Furthermore, cell-mediated, drug response is dependent on the cellular characteristic and the drug action. The formation of apoptotic bodies of the tested cells in response to the three used drugs differs among them (Figure 1(c)). For example, MCF-7 cells formed less and small apoptotic bodies in response to both Carboplatin and Oxaliplatin but not Cisplatin while BT-474 cells response was maximal for Carboplatin and MDA-MB-231 cells response was similar for all drugs.

### 3.3. PBDs-Induced Effects on the Intracellular Organelles of Breast Cancer Cells

TEM is an excellent tool that provides a qualitative bidimensional image of the intercellular organelles at high magnification. Therefore, in order to gain more insight into the ultrastructural alterations induced by PBDs and how the drug cytotoxicity differentially caused these alterations, the cells were prepared to be screened by TEM as mentioned above. Our findings demonstrated similar effects of PBDs that were shared among the tested cells in addition to distinct ones that were specific to each cell type that will be described later. Generally, the platinum deposits were detected at both concentrations and time points in the treated cells but not in the control (Figures 3(a), 4(a), and 5(a)). The deposits were scattered at different cellular compartments such as the cytoplasm (endoplasmic reticulum, mitochondria, and Golgi) and across its membrane as well as in the nucleus and through its envelope causing structural changes.

**Figure 2:**
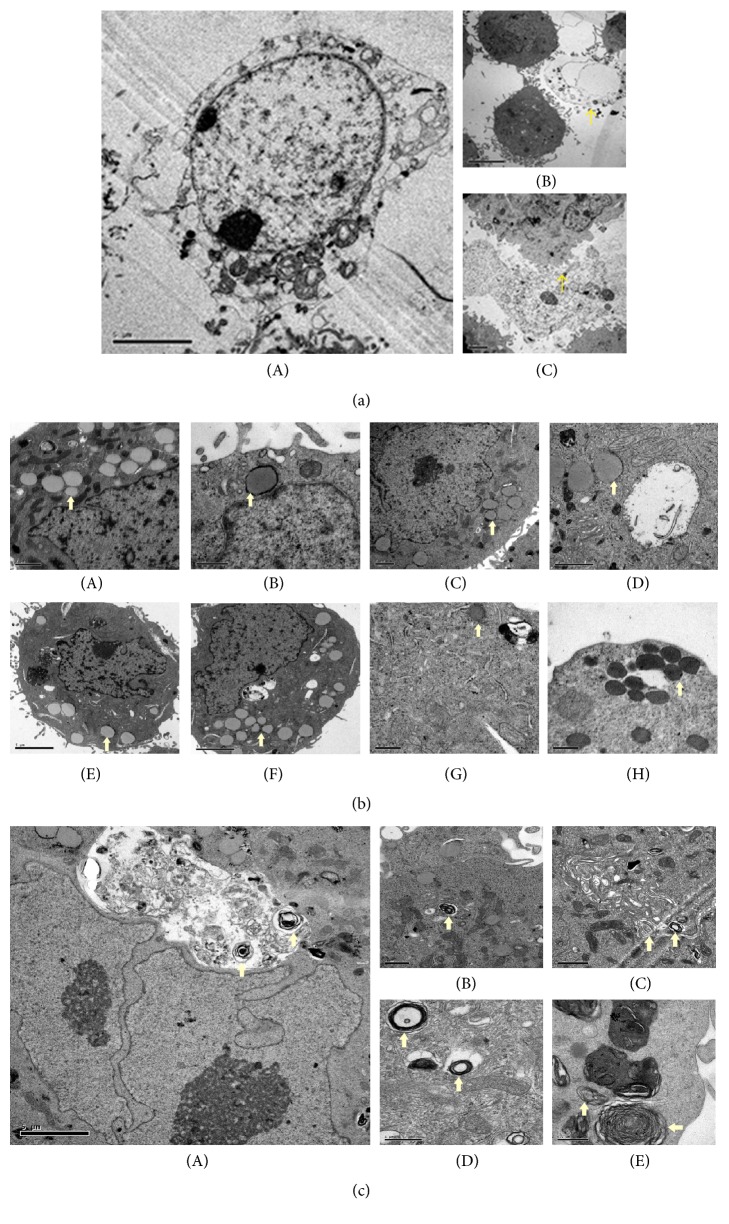
The effect of PBDs on the ultrastructure of breast cancer cells by TEM. (a) TEM micrographs representing necrosis of posttreated cancer cells with PBDs. (b) TEM micrographs illustrating PBDs deposit around and within the fat droplets of cancer cells. (A) Fat droplet in untreated cells. (B) MCF-7 treated with Cisplatin at 10 *μ*m for 2 h (50000x). (C) MCF-7 treated with Carboplatin at 20 *μ*m for 2 h (15000x). (D) MCF-7 treated with Oxaliplatin at 20 *μ*m for 2 h (30000x). (E) BT-474 cells treated with Cisplatin 20 *μ*m for 4 h (12000x). (F) BT-474 treated with Oxaliplatin at 20 *μ*m for 2 h (12000x). (G) MDA-MB-231 treated with Oxaliplatin at 10 *μ*m for 2 h (40000x). (H) MDA-MB-231 treated with Carboplatin at 10 *μ*m for 2 h (40000x). (c) TEM micrographs illustrating myelinated bodies observed on PBDs-treated cells (arrows). (A) and (B) Carboplatin treated MCF-7 at 10, 4 h (10000x). (C) BT-474 treated with Cisplatin at 20, 4 h (25000x). (D) BT-474 treated with Carboplatin at 10, 2 h (60000x). (E) MDA-MB-231 treated with Carboplatin at 10 for 4 h (50000x).

**Figure 3:**
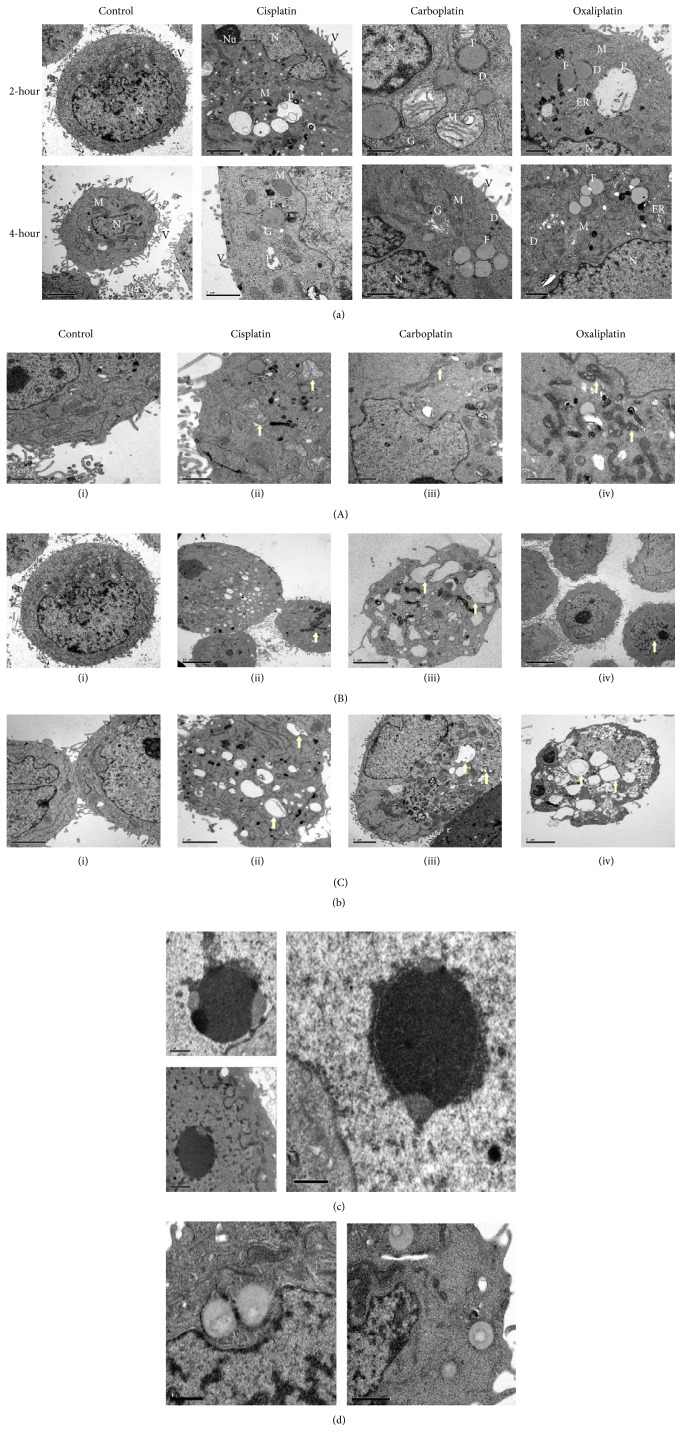
TEM micrographs of PDBs effect on MCF-7 cell line. (a) illustrates the effect of PBDs on the intracellular organelles of MCF-7 cells. PBDs deposits (D) were scattered over the intercellular organelles (N; nucleus, Nu; nucleolus, F; fat droplet, M; mitochondria, ER; endoplasmic reticulum, G; Golgi apparatus, P; phagosome, and V; villi) causing structural changes. Micrographs are screened at scale of 1–10 *μ*M. (b) MCF-7 treated cells with PBDs compared to control. (A) illustrates the ultrastructural change of mitochondria (arrows), (A)(i) untreated (20000x), (A)(ii) 10 *μ*M Cisplatin treated cell for 2 h (25000x), (A)(iii) 10 *μ*M Carboplatin treated cells for 4 h (20000x), and (A)(iv) 20 *μ*M Oxaliplatin treated cell for 4 h (25000x). (B) illustrates chromatin condensation and fragmentation (arrows), (B)(i) untreated (4000x), (B)(ii) 10 *μ*M Cisplatin treated cells for 4 h (5000x), (B)(iii) 20 *μ*M Carboplatin treated cells for 2 h (12000x), and (B)(iv) 20 *μ*M Oxaliplatin treated cell for 2 h (5000x). (C) demonstrates phagosome formation (arrows), (C)(i) untreated (12000x), (C)(ii) 20 *μ*M Cisplatin treated cells for 2 h (12000x), (C)(iii) 10 *μ*M Carboplatin treated cells for 2 h (8000x), (C)(iv) 10 *μ*M Oxaliplatin treated cell for 4 h (10000x). Micrographs are screened at scale of 1–10 *μ*M. (c) MCF-7 treated cells with 20 *μ*M of Oxaliplatin for 4 h showing specific characteristics of nucleolus (cap appearance). (d) MCF-7 treated cells with Oxaliplatin demonstrating specific feature of fat droplet (arrow).

**Figure 4:**
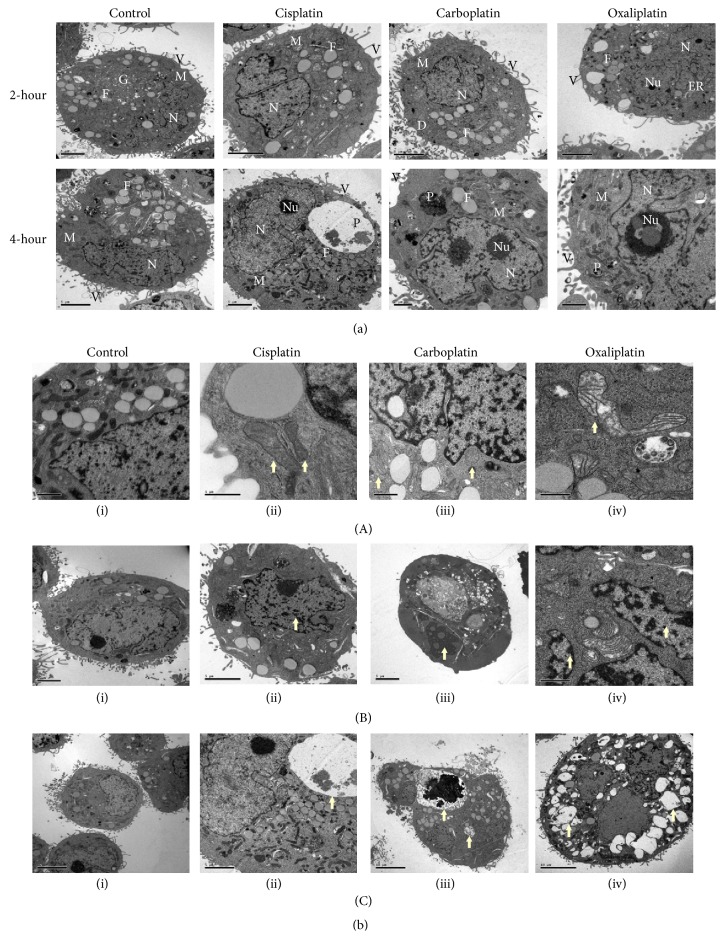
TEM micrographs of PDBs effect on BT-474 cell line. (a) illustrates the effect of PBDs on the intracellular organelles of BT-474 cells. PBDs deposits (D) were scattered over the intercellular organelles (N; nucleus, Nu; nucleolus, F; fat droplet, M; mitochondria, ER; endoplasmic reticulum, G; Golgi apparatus, P; phagosome, and V; villi) causing structural changes. (b) BT-474 treated cells with PBDs compared to control. (A) illustrates the ultrastructural change of mitochondria (arrows), (A)(i) untreated (20000x), (A)(ii) 10 *μ*M Cisplatin treated cell for 2 h (60000x), (A)(iii) 20 *μ*M Carboplatin treated cells for 2 h (80000x), and (A)(iv) 10 *μ*M Oxaliplatin treated cell for 2 h (50000x). (B) illustrates chromatin condensation and fragmentation (arrows), (B)(i) untreated (8000x), (B)(ii) 20 *μ*M Cisplatin treated cells for 4 h (8000x), (B)(iii) 20 *μ*M Carboplatin treated cells for 4 h (8000x), and (B)(iv) 10 *μ*M Oxaliplatin treated cell for 2 h (25000x). (C) demonstrates phagosome formation (arrows), (C)(i) untreated (5000x), (C)(ii) 20 *μ*M Cisplatin treated cells for 4 h (10000x), (C)(iii) 20 *μ*M Carboplatin treated cells for 4 h (5000x), and (C)(iv) 10 *μ*M Oxaliplatin treated cell for 2 h (6000x).

**Figure 5:**
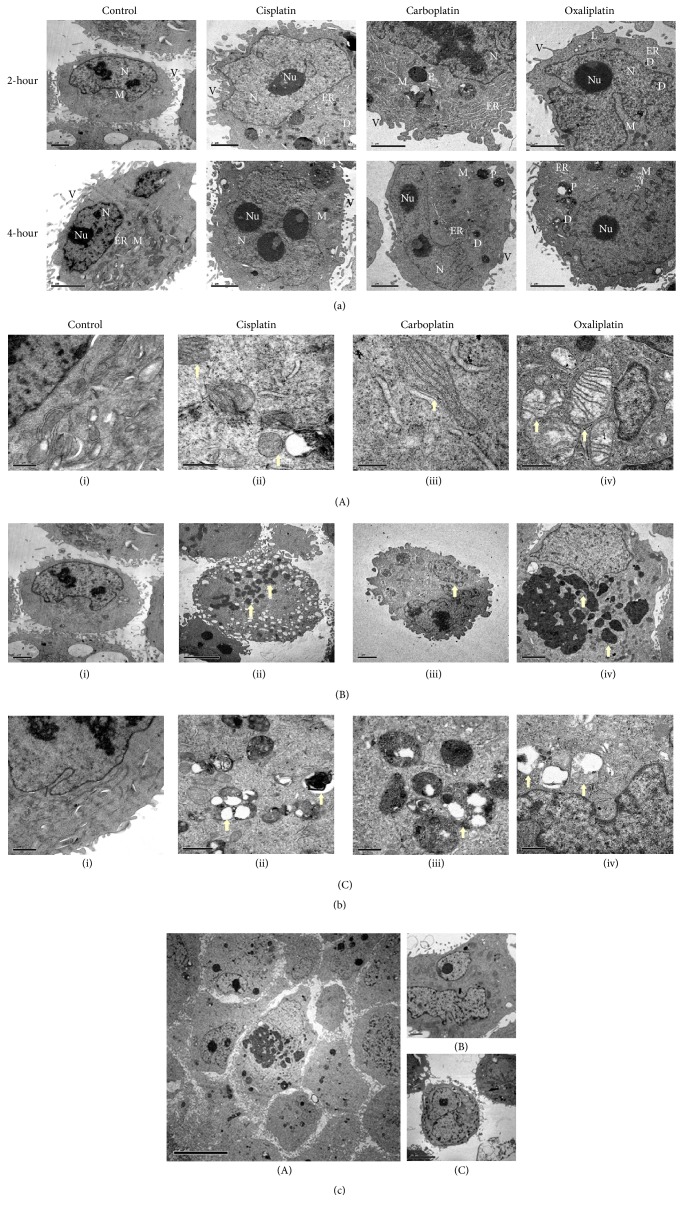
TEM micrographs of PDBs effect on MDA-MB-231 cell line. (a) TEM micrographs illustrating the effect of PBDs on the intracellular organelles of MDA-MB-231 cell line. PBDs deposits (D) were scattered over the intercellular organelles (N; nucleus, Nu; nucleolus, F; fat droplet, M; mitochondria, ER; endoplasmic reticulum, G; Golgi apparatus, P; phagosome, and V; villi) causing structural changes. (b) TEM micrographs of MDA-MB-231 treated cells with PBDs compared to control. (A) illustrates the ultrastructural change of mitochondria (arrows), (A)(i) untreated (40000x), (A)(ii) 10 *μ*M Cisplatin treated cell for 2 h (60000x), (A)(iii) 20 *μ*M Carboplatin treated cells for 4 h (120000x), and (A)(iv) 20 *μ*M Oxaliplatin treated cell for 4 h (50000x). (B) illustrates chromatin condensation and fragmentation (arrows), (B)(i) untreated (8000x), (B)(ii) 10 *μ*M Cisplatin treated cells for 4 h (6000x), (B)(iii) 10 *μ*M Carboplatin treated cells for 2 h (8000x), and (B)(iv) 10 *μ*M Oxaliplatin treated cell for 4 h (10000x). (C) demonstrates phagosome formation (arrows), (C)(i) untreated (20000x), (C)(ii) 10 *μ*M Cisplatin treated cells for 2 h (25000x), (C)(iii) 20 *μ*M Carboplatin treated cells for 2 h (40000x), and (C)(iv) 20 *μ*M Oxaliplatin treated cell for 2 h (40000x). (c) TEM micrographs represent some cells of MDA-MB-231 with no nucleus (A) while MCF-7 and BT-474 have more than one nucleus (B and C, resp.).

TEM micrographs of treated cells revealed two forms of cell death, the apoptotic and the necrotic death, each of which had specific features. The main detected alterations in regard to the apoptotic death was presented by the cell nucleus. Chromatin margination and compaction towards the nuclear periphery generate numerous compact electron dense micronuclei that are released in the extracellular space (Figures 3(b)(B), 4(b)(B), and 5(b)(B)). Cytosol condensation and blebbing were also found and classified to the apoptotic phenotype (Figures 3(b)(B)(iii), 3(b)(C)(iv), 4(b)(B)(iii), 5(b)(B)(ii), and 5(b)(B)(iii)). Furthermore, cell splitting in a number of apoptotic bodies usually characterizes the final stage of apoptosis. Occasionally, apoptotic cells, in vitro, undergo a late process of secondary necrosis. Necrosis is considered to be the messy way of cell death by which the early changes can be identified on plasma membrane that shows incoherence, causing cell swelling and organelles disruption (Figure 2(a)).

Interestingly, the deposit of PBDs was attracted around and within the fat droplet of treated cells compared to the fat droplets of untreated cells (Figure 2(b)). Another observation was the obvious lamellar bodies present in the treated cells as shown in Figure 2(c). These are specialized lipid storage or secretory organelles having a core composed of multilamellar structure and can be surrounded by a membrane [23]. Carboplatin treated cells exhibited more of the lamellar bodies compared to other PBD-treated cells.

### 3.4. PBDs-Mediated Alterations in MCF-7 Breast Cancer Cells

The ultrastructures of MCF-7 cells were distinct. The untreated cells showed apparent fat droplets, clear microvilli, and low number of Golgi apparatuses. Also, some lysosomes were present. PBDs-treated MCF-7 cells, after 2 hours, demonstrated drug accumulation around and within the fat droplets. Moreover, many phagosomes and big vacuoles were present (Figure 3(a)). At 4-hour period, most of the deposits were detected close to the cell membrane and outside the cell, indicating the active process of drug efflux. In addition, shorter microvilli and bigger vacuoles were also detected, some of which are characterized by double membrane (Figure 3(b)). Swelling and disarrangement of cell organelles such as mitochondria, endoplasmic reticulum, and Golgi apparatus were present (Figures 3(a) and 3(b)). Cisplatin and Carboplatin disrupted mitochondria by dilation while Oxaliplatin did that by disarrangement of its internal folds (Figure 3(b)(A)). At nuclear level, chromatin was condensed as dark inclusions (heterochromatin). Fragmentation of the nucleus was observed in some of the cells indicating the late stage of apoptosis (Figure 3(b)(B)). Oxaliplatin affected the nucleus by forming a cap appearance due to chromatin clumping showing brighter edges compared to its interior (Figure 3(c)). Moreover, Oxaliplatin affected the fat droplets by forming specific feature of a pale circle in their center (Figure 3(d)) unlike the other two drugs that accumulated around them.

### 3.5. PBDs-Mediated Alterations in BT-474 Breast Cancer Cells

TEM micrographs of control BT-474 cells, in comparison to MCF-7, revealed more fat droplets that were more uniform in size and color but they were bigger and fainter. However, microvilli were less in number which is consistent with the SEM micrograph (Figure 1(b)). The treated BT-474 cells had similar alterations to MCF-7 treated cells. Shrinkage of the cytoplasm with disappearance or retraction of the microvilli was observed (Figures 4(a) and 4(b)). The mitochondria in the treated cells were clumped compared to the nontreated cells in which they were spread over the cytoplasm. Swelling of mitochondria was observed when the cells were treated with Cisplatin whereas internal cristae of mitochondria were disarranged when they were treated with Oxaliplatin (Figure 4(b)(A)). On the other hand, no apparent effect was observed on the mitochondria when Carboplatin was used. PBDs-treated cells showed a double-membrane phagosome within some of the destructed organelles as shown in Figure 4(b)(C) while Figure 4(b)(B) demonstrated condensation and fragmentation of the nucleus accompanied by a decrease in cytoplasm to nucleus ratio. Moreover, Oxaliplatin resulted in chromatin clumping which gave it a cup shape appearance (Figure 4(a)).

### 3.6. PBDs-Mediated Alterations in MDA-MB231 Breast Cancer Cells

The MDA-MB-231 cells are the triple-negative cells that lack the three receptors (ER, PR, and Her2) and therefore have distinct features unlike the above two cells which at least have two receptors (MCF-7) out of the three or have them all (BT-474). The TEM micrographs revealed that these cells have very few fat droplets and more microvilli compared to MCF-7 and BT-474 cells as illustrated in Figure 5(a). Interestingly, most of these cells were detected without nucleus whereas the other cells had more than one (Figure 5(c)). Treated cells exhibited similar ultrastructural changes as treated MCF-7 and BT-474 cells. Microvilli of PBDs-treated cells were shortened and enlarged. Both Cisplatin and Oxaliplatin caused swollen mitochondria in these cells unlike the Carboplatin. Double-membrane phagosomes were present containing PBDs deposits and some of the destroyed organelles. Condensation and fragmentation of the nucleus were also observed with pale-circled nucleoli as demonstrated in Figures 5(a) and 5(b), respectively.

## 4. Discussion

The current study represents a comprehensive comparison of the effects of three generations of commonly used PBDs on three breast cancer cells representing the most diagnosed types. Although Cisplatin, Carboplatin, and Oxaliplatin shared similar effects on the ultrastructure of the three breast cancer types, specific alterations and responses were also demonstrated, supporting the differences related to each cell type and drug action.

SEM micrographs illustrated specific surface morphology for each tested cell type, confirming their origin and characteristics. Cancer cells gain new cellular features in order to survive, one of which is the formation of unique surface protrusions which is important to enhance movement and adhesion to the surrounding stroma unlike the normal breast cell (Figure 1(a)). Advancing cancer cells, such as MDA-MB231 with metastatic characteristics presented by high number of these lamellipodia, indicate the importance of shape modifications in their invasiveness process. Lamellipodia consist of protrusive filamentous actin and signaling proteins which play a role in cell migration and cell-cell communication [24]. These distinct features of triple-negative breast cancer cells in vivo model might demonstrate its aggressiveness and give them a metastatic potential [24–26]. As a result, treatment of MDA-MB-231 with PBDs led to retraction of microvilli and lamellipodia, which is one way to inhibit metastasis [27]. This apoptotic feature was also detected in MCF-7 and BT-474 treated cells but to a lesser extent.

The PBDs-mediated changes on the cell surface appeared within few minutes as illustrated by SEM, supporting the initial response of breast cancer cells, followed by several sequential events. These changes started with the formation of pores on the cell membranes, indicating the active process of drug influx/efflux. Subsequently, the lamellipodia of the PBDs-treated cells became retracted, causing them to shrink and thus having a semioval to round shape. Later on-set changes after prolonged treatment revealed the early stage of apoptosis presented by convoluted membrane, membrane blebs, and apoptotic bodies. The membrane blebbing is considered to be a specific pattern of apoptosis that is due to a deep cytoskeleton rearrangement, resulting in alterations of cell shape and organelles distribution. Although the above PBDs-mediated alterations were consistently detected in all tested cells, the original cell shape influences the appearance of these alterations. For example, the pores on the surface of MDA-MB 231 appeared to be deeper and wider due to the high number of membrane ruffles unlike the other two cell types, MCF-7 and BT-474, where the pores appeared smaller and narrower (Figure 1(b)). This might be true, suggesting that the PBDs caused pores might differ in size or shape depending on the exterior of the cells.

Furthermore, cell-mediated, drug response is dependent on the cellular characteristic and the drug action. The formation of apoptotic bodies of the tested cells in response to the three used drugs differs among them (Figure 1(c)). For example, MCF-7 cells formed few and small apoptotic bodies in response to both Carboplatin and Oxaliplatin but not Cisplatin while BT-474 cells response was maximum for Carboplatin and MDA-MB231 cells response was similar for all drugs.

The distinct morphological characteristics of cell death observed by TEM such as shrinkage of cytoplasm, microvilli retraction, fragmentation and condensation of nucleus, and swelling of some organelles such as mitochondria and endoplasmic reticulum suggest that the cells commit cell death mainly via apoptosis [27]. These apoptotic findings were consistent with other findings in which apoptosis was the major mechanism of cell death that appeared in MCF-7 cells following exposure to Cisplatin [18, 28]. Interestingly, the absence of nucleus in MDA-MB-231 after treatment with PBDs was observed which might be explained by the extrusion of nucleus as a way for cancer cells to die. An in vitro conditions-enucleation in MCF-7 breast cancer cells was performed by Paunescu et al., showing that cancer cells were stressed and died compared to normal cells which are not affected [29]. Independently of mediating apoptosis through nuclear pathway, PBDs have been found to activate caspases through endoplasmic reticulum stress. This can be attributed to different cellular pathways including the activation of Ca^2+^-dependent calpain proteases which activate caspase-3. Also, it has been found that Cisplatin has an effect on the regulation of cyclin-dependent kinase- (Cdk2-) E2F1 pathway. Cdk1 complex is located in the ER leading to its stress [30]. We recently showed that Cisplatin induces apoptosis through the endoplasmic reticulum-mediated, calpain 1 pathway in MDA-MB-231 cells. In this study, we demonstrated that the ultrastructure analysis of endoplasmic reticulum correlated with the level of apoptosis caused by Cisplatin, clearing any discrepancies between the two events [31].

Our findings also demonstrated a distinct feature which is the increased number of vacuoles as a defending mechanism of cell survival caused by PBDs-dependent stress which is consistent with the literature [32–35]. These vacuoles are known as the double-membranous autophagosome and autolysosomes that usually contain destructed organelles and PBDs deposits [36].

The platinum deposits were detected scattering at different cellular compartments at both concentrations (10 and 20 *μ*M) and time points (2 and 4 hours). This suggests the possible use of PBDs by the proposed timing and concentrations. Interestingly, the drugs deposits were mainly attracted to the fat droplets of the cells, suggesting an active role of cellular lipids in potentiation of PBDs treatment and induction of apoptosis. Some studies found that the bioactive lipid molecules such as polyunsaturated fatty acids (PUFA) play a role in cell signaling proliferation and cell death [37–39]. A study by Zajdel et al. found that the PUFA, eicosapentaenoic acid, and docosahexaenoic acid increased the antitumor activity of Cisplatin in A549 human lung adenocarcinoma cells [40]. Other lipid molecules such as apolipoproteins, cholesterol, phosphatidic acid, and ceramide can induce apoptosis through modulating mitochondrial membrane permeability and activating different enzymes including caspases [41]. Another observation related to lipid structure is the lamellated membrane structures representing cell stress due to toxic drug effect. It is possible that PBDs induce lipidosis in cancer cells and cause accumulation of lamellar bodies [23].

## 5. Conclusions

The findings of this study presented for the first time a comprehensive comparison of cellular features of representative breast cancer cells treated with three commonly used PBDs. The results revealed that breast cancer cells exhibited a differential response to the various drugs, directing further investigations to purse specific pathways in determining breast cancer cells sensitivity to PBDs.

## Abbreviations

CO_2_: Carbon dioxide
DMEM: Dulbecco's Modified Eagle Medium
DNA: Deoxyribonucleic acid
EDTA: Ethylenediaminetetraacetic acid
ER: Estrogen receptors
HER2: Human epidermal growth factor receptor 2
HMDS: Hexamethyldisilazane
NCBI: National Cell Bank of Iran
OS: Overall survival
PBDs: Platinum-based drugs
PBS: Phosphate buffer saline
PgR: Progesterone receptors
PUFA: Polyunsaturated fatty acids
RPMI: Roswell Park Memorial Institute
SEM: Scanning electron microscopy
TEM: Transmission electron microscopy
TNBC: Triple-negative breast cancer.
